# Involvement of RORγt-overexpressing T cells in the development of autoimmune arthritis in mice

**DOI:** 10.1186/s13075-015-0606-5

**Published:** 2015-04-20

**Authors:** Yuya Kondo, Zhaojin Yao, Masahiro Tahara, Mana Iizuka, Masahiro Yokosawa, Shunta Kaneko, Seiji Segawa, Hiroto Tsuboi, Keigyou Yoh, Satoru Takahashi, Isao Matsumoto, Takayuki Sumida

**Affiliations:** Department of Internal Medicine, Faculty of Medicine, University of Tuskuba, 1-1-1 Tennodai, Tsukuba City, Ibaraki 305-8575 Japan; Department of Anatomy and Embryology, Faculty of Medicine, University of Tsukuba, 1-1-1 Tennodai, Tsukuba City, Ibaraki 305-8575 Japan; International Institute for Integrative Sleep Medicine (WPI-IIIS), University of Tsukuba, 1-1-1 Tennodai, Tsukuba City, Ibaraki 305-8575 Japan; Life Science Center, Tsukuba Advanced Research Alliance (TARA), University of Tsukuba, 1-1-1 Tennodai, Tsukuba City, Ibaraki 305-8575 Japan; Laboratory Animal Resource Center (LARC), University of Tsukuba, 1-1-1 Tennodai, Tsukuba City, Ibaraki 305-8575 Japan

## Abstract

**Introduction:**

Differentiation of T helper 17 cells is dependent on the expression of transcription retinoid-related orphan receptor gamma t (RORγt). The purpose of our study is to determine the role of RORγt expression in T cells on the development of collagen-induced arthritis (CIA).

**Methods:**

CIA was induced in C57BL/6 and T cell-specific RORγt transgenic (RORγt Tg) mice. At day 10 post-1st-immunization, lymph node (LN) cells were cultured with type II collagen (CII), and the expression levels of various cytokines and transcription factors on CD4^+^ T cells were measured. Total cells or CD4^+^ cells of draining LN were harvested from each mouse group after CII-immunization and transferred into C57BL/6 mice, and then CIA was induced in recipient mice. The expression levels of RORγt and other surface antigens, and the production of cytokines were analyzed in forkhead box P3 (Foxp3)^+^ regulatory T (Treg) cells. Foxp3^+^ Treg cells were analyzed for suppressive activity against proliferation of effector CD4^+^ T cells. Interlukin (IL)-10 neutralizing antibody was administrated in the course of CIA.

**Results:**

CIA was significantly suppressed in RORγt Tg mice compared with C57BL/6 mice. RORγt expression and IL-17 production were significantly higher in CII-reactive CD4^+^ T cells from RORγt Tg mice. Arthritis was significantly attenuated in C57BL/6 mice recipient of cells from RORγt Tg mice. Most of Foxp3^+^ Treg cells expressed RORγt, produced IL-10 but not IL-17, and overexpressed CC chemokine receptor 6 (CCR6) and surface antigens related to the suppressive activity of Foxp3^+^ Treg cells in RORγt Tg mice. In vitro suppression assay demonstrated significant augmentation of the suppressive capacity of Foxp3^+^ Treg cells in RORγt Tg mice. CIA was exacerbated in both C57BL/6 mice and RORγt Tg mice by the treatment of anti-IL-10 antibody.

**Conclusion:**

Our results indicated that RORγt overexpression in T cells protected against the development of CIA. The protective effects were mediated, at least in part, through the anti-inflammatory effects including high production of IL-10 of RORγt^+^Foxp3^+^ Treg cells.

## Introduction

Rheumatoid arthritis (RA) is a chronic inflammatory disorder characterized by autoimmunity, infiltration of activated inflammatory cells into the joint synovium, synovial hyperplasia, neoangiogenesis, and progressive destruction of the cartilage and bone. This disease affects 1 to 2% of the population worldwide, most commonly middle-aged women. The etiology of RA is unknown but pro-inflammatory cytokines seem to play a central role. Thus, correction of any cytokine imbalance can probably control this disease.

T cells form a large proportion of the inflammatory cells invading the synovial tissue. CD4^+^ T cells are one of the T cell subsets involved in the RA pathological process. Upon antigenic stimulation and cytokine signaling, naïve CD4^+^ T cells activate and differentiate into various T helper (Th) subsets [1]. Classically Th cells are divided into Th1 and Th2 subsets according to their cytokine production pattern. Recently, IL-17-producing Th17 cells have been identified and this T cell population appears to play a critical role in the generation of several types of autoimmune arthritis such as glucose-6-phosphate isomerase (GPI)-induced arthritis [2] and collagen-induced arthritis (CIA) [3]. Moreover, blockade of IL-17 after disease onset prevents cartilage and bone destruction, leading to amelioration of the clinical symptoms of the disease in CIA [4]. Another study identified IL-17 receptor signaling as a critical pathway in turning acute synovitis into chronic destructive arthritis [5]. In RA patients, IL-17 is spontaneously produced by the rheumatoid synovium [6], and a high percentage of IL-17-positive CD4^+^ T cells in peripheral blood mononuclear cells have been detected in RA patients compared with healthy control subjects [7]. Therefore, Th17 is considered to be related to the development of RA. Lineage commitment of each Th cell subset from naive CD4^+^ T cells is dependent on the expression of specific transcription factors induced by specific cytokine environment. Each Th cell-specific transcription factor does not only regulate the expression of effector molecules like cytokines and chemokines specific for each Th cell subset, but also negatively regulates the differentiation of other T cell subsets [8,9].

Differentiation of Th1 and Th2 cells is dependent on the expression of transcription factor T-box transcription factor (T-bet) [10] and GATA binding protein-3 (GATA-3) [11], respectively. Similarly, transforming growth factor-β (TGF-β) and IL-6 induce the expression of the transcription factor RORγt, which upregulates the expression of Th-17-specific molecules, IL-17A, IL-17 F, CC chemokine ligand 20 (CCL20), and chemokine receptor CCR6 in mice [12-14]. Recent studies highlighted the importance of Th cell-specific transcription factors in the development of autoimmune arthritis. For example, in mice models of autoimmune arthritis, GATA-3 expression protects against joint inflammation and destruction by reducing the differentiation of Th17 cells [15]. Furthermore, we reported previously that T-bet expression regulates the development of autoimmune arthritis by suppression of antigen reactive Th17 cells differentiation via interferon (IFN)γ-independent suppression of RORγt expression [16]. In RA patients, CD4^+^ T cells overexpress IL-17 and RORC (encoding RORγt), compared with healthy control subjects [17]. Thus, more work is needed to determine whether RORγt expression and dominant differentiation of Th17 play a role in the development of autoimmune arthritis.

Recently, Yoh *et al*. [18] reported that the T cells of RORγt transgenic (RORγt Tg) mice under the control of CD2 promoter express high levels of RORγt and exhibit a dominant Th17 differentiation pattern [18]. In the present study, CIA was induced in both RORγt Tg mice and C57BL/6 mice. The results showed significant protection of RORγt Tg mice against experimentally induced CIA compared with C57BL/6 mice. Furthermore, although RORγt expression and IL-17 production in type II collagen (CII)-reactive CD4^+^ T cells were significantly higher in RORγt Tg mice, arthritis was significantly attenuated in C57BL/6 mice recipients of cells from immunized RORγt Tg mice in adoptive transfer of draining lymph node (LN) cells or recipients of CD4^+^ cells. Foxp3^+^ Treg cells overexpressed RORγt and CCR6, produced IL-10 but not IL-17, and preferentially infiltrated into the joints of RORγt Tg mice, compared with C57BL/6 mice, after the induction of CIA. *In vitro* suppression assay demonstrated that the suppressive activity of Foxp3^+^ Treg cells was significantly augmented in RORγt Tg mice compared with C57BL/6 mice. CIA was significantly exacerbated in RORγt Tg mice by the administration of neutralizing antibody of IL-10. Our results suggest that the inhibition of arthritis in RORγt Tg mice was mediated by suppressor cell subsets, including IL-10 producing CCR6^+^RORγt^+^Foxp3^+^ Treg cells.

## Methods

### Mice

Age- and sex-matched C57BL/6 mice and C57BL/6 CD2- RORγt Tg mice (age 6 to 10 weeks) were used in our experiments. RORγt Tg mice were prepared by backcrossing mice on the C57BL/6 background. For the isolation of Treg cells, RORγt Tg and C57BL/6 mice were crossed with knockin mice with Foxp3-IRES-green fluorescent protein (GFP) (C57BL/6-Foxp3^GFP^ and RORγt Tg-Foxp3^GFP^ mice) provided by B Malissen (*Université de la Méditerranée*, Marseille, France). All mice were maintained under specific pathogen-free conditions. All experiments described in this report were performed according to the Guide for the Care and Use of Laboratory Animals at the University of Tsukuba, and were approved by the Animal Ethics Review Committee of the University of Tsukuba.

### Induction of collagen-induced arthritis

Native chicken CII was obtained from Sigma-Aldrich (St Louis, MO, USA). CII was dissolved in 0.01 M acetic acid and emulsified in complete Freund’s adjuvant (CFA). CFA was prepared by mixing 5 mg heat-killed *Mycobacterium tuberculosis* (H37Ra; Difco Laboratories, Detroit, MI, USA) and 1 mL incomplete Freund’s adjuvant (Sigma-Aldrich). Mice were immunized intradermally at the base of the tail with 200 μg CII in CFA on days 0 and 21. Arthritis was evaluated visually, and changes in each paw were scored on a scale of 0 to 3 as follows; 0 = normal, 1 = slight swelling and/or erythema, 2 = pronounced swelling, 3 = ankylosis. The score was summed for each limb (maximum score = 12). For histological assessment, mice were sacrificed at day 42 post first CII immunization, and both hind limbs were removed. After fixation and decalcification, the joints were cut into sections and stained with hematoxylin and eosin. Quantification of histological changes was carried out by two independent and blinded observers, and a histological score was assigned to each joint based on the degree of inflammation and erosion, as described previously [16,19]. In the experiment of IL-10 neutralization in the course of CIA, 100 μg of anti-IL-10 antibody (JES5-16E3; BioLegend, San Diego, CA, USA) or isotype control antibody (RTK4530; BioLegend) was administrated intraperitoneally every 2 days from day 22 to 30 post first CII immunization.

### Cell isolation

Inguinal lymph nodes were collected as draining LNs, and used for the experiments. CD4^+^ cells in draining LNs and spleen were isolated by positive selection using a magnetic-activated cell sorting (MACS) system with anti-CD4 mAb (Miltenyi Biotec, Bergisch Gladbach, Germany). CXC chemokine receptor 5^+^ (CXCR5) follicular helper T (Tfh) cells were isolated with Moflo cell sorter (DakoCytomation, Glostrup, Denmark) from MACS-isolated CD4^+^ T cells. For the isolation of Foxp3^+^ Treg cells and Foxp3^−^ non-Treg cells, CD4^+^GFP^+^ and CD4^+^GFP^−^ cells were further purified using a Moflo cell sorter from MACS-isolated CD4^+^ T cells in C57BL/6-Foxp3^GFP^ and RORγt Tg-Foxp3^GFP^ mice. The isolated cells were used for the experiment of adoptive cell transfer and quantitative reverse transcriptase-polymerase chain reaction (RT-PCR) (see below).

### Adoptive cell transfer experiment

All cells of draining LN and CD4^+^ cells were harvested from C57BL/6 mice and RORγt Tg mice at day 10 post first CII immunization. Cells were resuspended in PBS, and 1 × 10^7^ cells from the draining LNs or 2 × 10^6^ of CD4^+^ cells were injected intravenously into C57BL/6 mice at day 10 post first CII immunization. Recipient mice were immunized with CII in CFA intradermally on day 11 after the cell transfer. Arthritis was evaluated visually as described above.

### Quantitative RT-PCR

Total RNA was prepared from Tfh cells isolated from draining LNs on day 10 post first CII immunization with RNeasy Plus Micro (QIAGEN, Venlo, Netherlands) according to the instructions provided by the manufacturer. cDNA was obtained by reverse transcription with a commercially available kit (TaKaRa Bio, Otsu, Japan). A TaqMan Assay-on-Demand gene expression product was used for real-time PCR (Applied Biosystems, Foster City, CA, USA). The expression levels of *IL21*and *Bcl6* were normalized relative to the expression of *GAPDH*. Analysis was performed with ABI Prism 7500 apparatus (Applied Biosystems).

### Cell culture

Draining LN cells were harvested from each mouse at day 10 post first CII immunization. Single cell suspension was prepared, and LN cells (4 × 10^5^ cells/well on a 96-well round-bottom plate) were cultured in Roswell Park Memorial Institute (RPMI) 1640 medium (Sigma-Aldrich) containing 10% FBS, 100 units/mL of penicillin, 100 μg/mL of streptomycin, and 50 μM 2-mercaptoethanol. The LN cells were cultured in the presence of 100 μg/mL denatured chicken CII for 72 h and analyzed for CII-reactive cytokine production. Furthermore, CD4^+^GFP^+^ were stimulated with Dynabeads Mouse T-activator CD3/CD28 (Invitrogen, Carlsbad, CA, USA) (1 bead/cell) in round-bottomed 96-well dishes for 96 h and the amount of IL-10 produced by Foxp3^+^ Treg cells was measured.

### Chemotaxis assay

Cell migration was evaluated using a 24-well, 3-μm pore-size Transwell system (Corning, Lowell, MA, USA). Briefly, 5 × 10^5^ or 2.5 × 10^5^ of CD4^+^ cells isolated from draining LN cells were placed on the top of the Transwell, while CCL20 was added to the bottom of the Transwell system with or without 10 μg/mL of anti-IL-10 mAb or isotype control antibody. After 4 h of incubation at 37°C, the number of cells that migrated into the lower well was counted by flow cytometry. Foxp3 expression in the isolated CD4^+^ cells was also analyzed by flow cytometry before and after the migration.

### Flow cytometry

For flow cytometry, the cell surface was stained with the following antibodies specific for mouse proteins: anti-CD4 (RM4-5 or GK1.5), anti-CD3 (145-2C11), anti-programmed cell death-1 (29 F.1A12), anti-inducible T-cell co-stimulator (ICOS) (C398.4A), anti-CC chemokine receptor 6 (CCR6) (29–2 L17), anti-cytotoxic T-lymphocyte-associated protein 4 (CTLA4) (UC10-4B9), anti-glucocorticoid-induced tumor necrosis factor receptor (GITR) (DTA-1; all from BioLegend) and anti-CXCR5 CXCR5 (2G8; BD PharMingen, San Diego, CA, USA). For intracellular cytokine staining, cells were cultured with or without CII for 72 h, and GolgiStop (BD PharMingen) was added during the last 4 h of each culture. The cell surface was stained and then permeabilized with Cytofix/Cytoperm solution (BD PharMingen). This was followed by intracellular cytokine staining with anti-IL-17A (TC11-18H10; BD PharMingen), anti-IFNγ (XMG1.2; BioLegend), and anti-IL-10 antibodies (JES5-16ES; BioLegend). Mouse Regulatory T Cell Staining Kit (eBioscience, San Diego, CA, USA) was used to stain the transcription factors with anti-Foxp3 (MF-14; Biolegend), anti-T-bet (eBio4B10) and anti-RORγt antibodies (AFKJS-9; both from eBioscience). Annexin V (BioLegend) and propidium iodide (PI) (BioLegend) are used for the detection of apoptotic cells. Data were acquired on a FACSCalibur flow cytometer (Becton Dickinson, Mountain View, CA, USA), and analyzed with FlowJo software (Tree Star, Ashland, OR, USA).

### Analysis of cytokine profiles

The supernatants were collected after the cells were cultured with or without CII for 72 h and the levels of IL-17, IFNγ, and IL-10 were analyzed by ELISA using the Quantikine ELISA kit (R&D Systems, Minneapolis, MN, USA).

### Measurement of collagen-specific IgG titers

Fifty-six days post first CII immunization, serum was collected, then diluted 1:4,000 in blocking solution containing 1% bovine serum albumin (Wako Pure Chemical Industries, Osaka, Japan) in PBS. Collagen-specific total IgG, IgG1, IgG2a, IgG2b, and IgG3 titers were measured by coated 10 μg/mL of CII in PBS on 96-well plates (Nunc Maxisorp; Nalge Nunc International, Roskilde, Denmark). The optical density was read at 450 nm using a microplate reader.

### *In vitro* suppression assay and Treg culture

Responder cells (CD4^+^CD25^−^GFP^−^) were labelled with 5 μM carboxyfluorescein diacetate succinimidyl ester (CFSE, Invitrogen), then cultured with or without unlabeled Treg cells (CD4^+^GFP^+^) at the indicated ratio for 96 h in the presence of Dynabeads Mouse T-activator CD3/CD28 (1 bead/cell) in round-bottomed 96-well dishes. The proliferation inhibition rate on the responder was calculated as: (1-(CFSE percentage Treg plus responder cells co-culture / responder cells alone)) X 100%.

$$ \left(1\hbox{--} \left(\mathrm{CFSE}\ \mathrm{percentage}\ \mathrm{Treg}\ \mathrm{plus}\ \mathrm{responder}\ \mathrm{c}\mathrm{ells}\ \mathrm{c}\mathrm{o}-\mathrm{culture}/\mathrm{responder}\ \mathrm{c}\mathrm{ells}\ \mathrm{alone}\right)\right) \times 100\%. $$

### Statistical analysis

Data are expressed at mean ± standard error of the mean (SEM). Differences between groups were examined for statistical significance using Student’s *t*-test. *P*-values <0.05 were considered significant.

## Results

### Suppression of CIA in RORγt Tg mice

**Figure 1:**
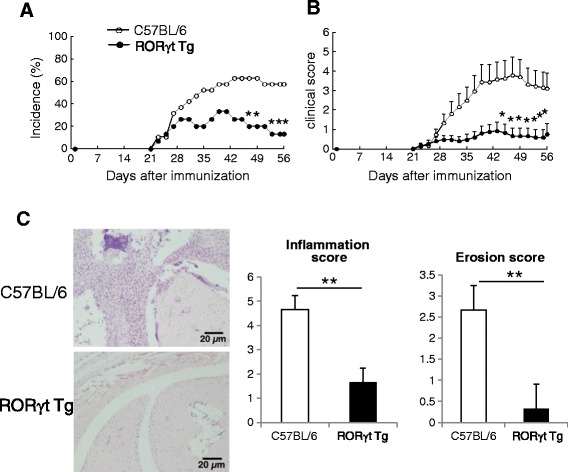
Significant suppression of collagen-induced arthritis (CIA) in RORγt Tg mice. Wild-type (WT) C57BL/6 mice (n = 19) and RORγt Tg mice (n = 15) were immunized intradermally with chicken type II collagen (CII) emulsified with complete Freund’s adjuvant (CFA) on days 0 and 21. **(A)** Incidence of arthritis. **(B)** Severity of CIA. Data obtained from three independent experiments. **(C)** At day 42 post first CII immunization, joint pathology was evaluated on decalcified hematoxylin and eosin-stained sections. Inflammation and bone erosion scores were assessed in both groups. Data are mean ± standard error of the mean. *P < 0.05, **P < 0.01, versus C57BL/6 mice, Student’s *t*-test.

To evaluate the effects of T cell-specific RORγt expression on the development of arthritis, we compared the severity of CIA in RORγt Tg mice and C57BL/6 mice. The incidence of arthritis in RORγt Tg mice was markedly suppressed compared with C57BL/6 mice (Figure 1A). Similarly, the arthritis score was also markedly lower in RORγt Tg than C57BL/6 mice (Figure 1B). Histological analysis of arthritic joints showed widespread infiltration of inflammatory cells and synovial hypertrophy throughout joint tissues of C57BL/6 mice, but not those of RORγt Tg mice (Figure 1C). Furthermore, analysis of joint inflammation and erosion scores showed significant suppression of inflammation and destruction in RORγt Tg mice compared with C57BL/6 mice (Figure 1C). These results indicate that RORγt Tg-expressing T cells suppressed the development of CIA.

### Overexpression of RORγt modulates differentiation of CII-reactive CD4^+^ T cells

**Figure 2:**
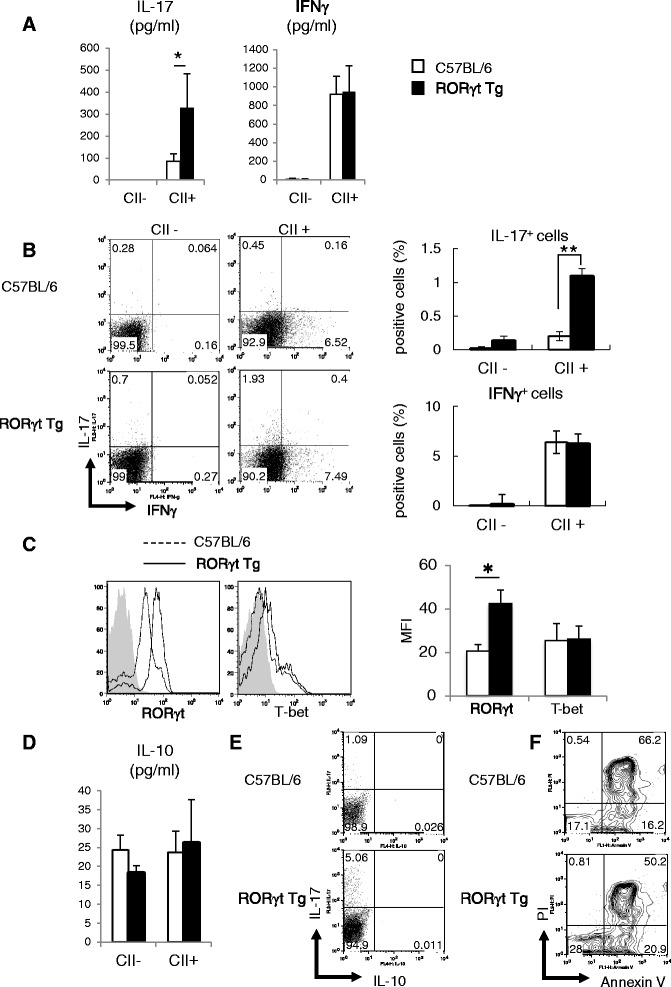
Cytokine production and transcription factor expression in antigen reactive CD4^+^ T cells. At day 10 days post first type II collagen (CII) immunization, draining lymph node (LN) cells from C57BL/6 mice (n = 3 or 4) and RORγt Tg mice (n = 3 or 4) were cultured with or without 100 μg/mL of denatured CII for 72 h. **(A)** IL-17 and IFNγ levels in supernatants measured by ELISA. **(B)** Cells were analyzed by staining for intracellular IL-17 and IFNγ. Data represent gated CD3^+^ and CD4^+^. Bar graphs show percentages of cytokine-positive cells. **(C)** The expression levels of RORγt and T-bet were evaluated by intracellular staining. Data represent gated CD3^+^ and CD4^+^. In the histograms, the gray shaded area represents data for the isotype control. Bar graphs show mean fluorescence intensity (MFI) of T-bet and RORγt Tg. **(D)** IL-10 levels in supernatants measured by ELISA. **(E** **,** **F)** Cells were analyzed by staining for intracellular IL-17 and IL-10 **(E)**, and propium iodide (PI) and Annexin V **(F)**. Data represent gated CD3^+^ and CD4^+^. Data are representative of two independent experiments with similar results, and are mean ± standard error of the mean. *P < 0.05, **P < 0.01, versus C57BL/6 mice, Student’s *t*-test.

To determine the effects of RORγt overexpression on CII-reactive cytokine production, we analyzed draining LN cells harvested at day 10 post first CII immunization. These cells were stimulated with CII *in vitro* before measurement of cytokine levels in supernatants by ELISA. The level of IL-17 was significantly higher in RORγt Tg mice than in C57BL/6 mice, whereas that of IFNγ was comparable in the two groups (Figure 2A). Fluorescence-activated cell sorting (FACS) analysis of cytokine expression on CD4^+^ T cells also showed no significant difference in IFNγ production from CII-reactive CD4^+^ T cells between the two groups of mice (Figure 2B). The same analysis showed a significantly higher IL-17 production by CII-reactive CD4^+^ T cells of RORγt Tg mice compared with C57BL/6 mice (Figure 2B). We measured the expression of transcription factors critical for differentiation of each CD4^+^ T cells subset. The mean fluorescence intensity (MFI) of RORγt in CII-reactive CD4^+^ T cells was significantly higher in RORγt Tg mice than C57BL/6 mice (Figure 2C), but there was no difference in T-bet expression between C57BL/6 and RORγt Tg mice (Figure 2C). We also measured IL-10 production by CII-reactive CD4^+^ T cells by ELISA and FACS. There was no significant difference in the level of IL-10 between the two groups of mice (Figure 2D), and IL-10 production by CII-reactive CD4^+^ T cells was almost undetectable with FACS analysis in both mice (Figure 2E). Finally, we examined whether overexpression of RORγt in T cells increases apoptosis in these cells. FACS analysis of apoptotic cells stained with PI and annexin V showed that there was no significant difference between the two groups of mice in the percentage of apoptotic cells in CD4^+^ T cells cultured with CII *in vitro* (Figure 2F). These results indicate that overexpression of RORγt induced higher IL-17 production from CII-reactive CD4^+^ T cells, but had no effect on Th1 differentiation and CII-reactive cytokine production other than IL-17,

### Suppression of anti-CII antibody formation

**Figure 3:**
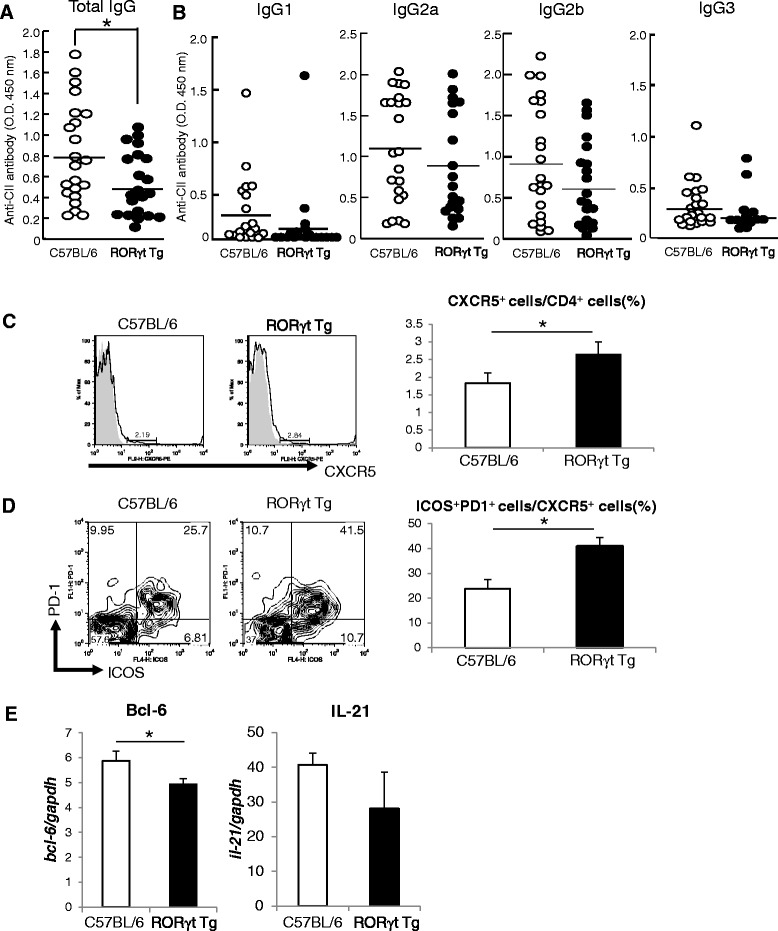
Suppression of autoantibody formation. **(A** **,** **B)** At day 56 post first type II collagen (CII) immunization, serum samples were collected from C57BL/6 mice (n = 22) and RORγt Tg mice (n = 21) for measurement of CII-specific total IgG levels **(A)**, and CII-specific IgG1, IgG2a, IgG2b, and IgG3 **(B)** by ELISA. Horizontal lines represent the mean values. **(C** **,** **D)** At day 10 post first CII immunization, the expression of CXCR5 **(C)** and the expression of inducible T-cell co-stimulator (ICOS) and programmed cell death-1 (PD-1) **(D)** in draining lymph nodes (LNs) were analyzed by fluorescence-activated cell sorting (FACS). Data represent gated CD4^+^ **(C)** and CXCR5^+^CD4^+^ cells **(D)**. Bar graphs show percentages of CXCR5^+^ cells **(C)** and ICOS^+^PD-1^+^ cells **(D)**. **(E)** cDNA was obtained from sorted CXCR5^+^CD4^+^ T cells isolated from draining LNs on day 10 post first CII immunization, and expression levels of Bcl-6 and IL-21 were analyzed by real-time PCR. Data are representative of two independent experiments with similar results, and represent the mean ± standard error of the mean. *P < 0.05, versus C57BL/6 mice, Student’s *t*-test.

We examined CII-specific IgG production in RORγt Tg mice, because CII-specific IgG level is known to correlate with the development of CIA [20]. Sera were collected at day 56 post first CII immunization, and the levels of CII-specific total IgG, IgG1, IgG2a, IgG2b, and IgG3 were measured by ELISA. Serum CII-specific total IgG was significantly lower in RORγt Tg mice than C57BL/6 mice (*P* <0.05, Figure 3A), but there was no significant difference in CII-specific IgG1, IgG2a, IgG2b and IgG3 (Figure 3B).

The next experiment examined whether the expression of RORγt affected the differentiation and function of T follicular helper (Tfh) cells. For this purpose, we measured the expression of CXCR5, programmed death-1 (PD-1), and inducible T-cell co-stimulator (ICOS), a marker of Tfh cells, by FACS analysis of CD4^+^ cells in draining LNs, the expression of B cell lymphoma 6 (Bcl-6), a master transcription factor of Tfh cell differentiation, and the expression of IL-21 in Tfh cells. The expression of Bcl-6 and IL-21 were conducted at day 10 post first CII immunization by real-time PCR using CXCR5^+^CD4^+^ Tfh cells from draining LNs sorted by FACS. The FACS analysis showed significantly higher expression of CXCR5 in CD4^+^ T cells in RORγt Tg mice than C57BL/6 mice (Figure 3C). In addition, ICOS^+^PD-1^+^ cells in CXCR5^+^CD4^+^ cells was significantly increased in RORγt Tg mice compared with C57BL/6 mice (Figure 3D). On the other hand, the expression level of Bcl-6 was significantly lower on CXCR5^+^CD4^+^ Tfh cells of RORγt Tg mice than that of C57BL/6 mice, and the expression levels of IL-21 on CXCR5^+^CD4^+^ Tfh cells also tended to be reduced in RORγt Tg compared with C57BL/6 mice (Figure 3E), suggesting the possibility that the differentiation and the function of Tfh cells is suppressed in RORγt Tg mice. These results indicate that suppression of CIA might be related to decreased anti-CII antibody formation caused by the dysfunction of Tfh cells in RORγt Tg mice.

### Transfer of T cells from RORγt Tg mice suppresses CIA in immunized C57BL/6 mice

**Figure 4:**
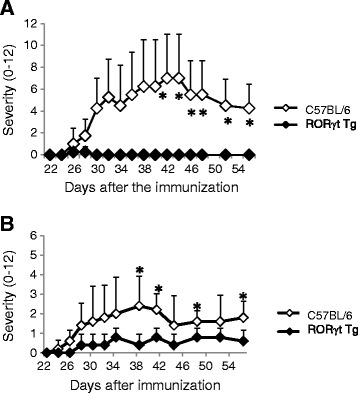
Suppression of collagen-induced arthritis (CIA) in mice after adoptive transfer of cells from RORγt Tg mice. **(A)** Severity scores of arthritis. Total draining lymph-node cells were harvested from C57BL/6 mice and RORγt Tg mice at day 10 post first type II collagen (CII) immunization, and 1 × 10^7^ cells were injected into C57BL/6 mice intravenously at day 10 post first CII immunization. Recipient mice were also immunized with CII in complete Freund’s adjuvant (CFA) intradermally on day 11 after the cell transfer. **(B)** CD4^+^ cells were isolated from draining lymph nodes of C57BL/6 mice and RORγt Tg mice at day 10 post first CII immunization, and 2 × 10^6^ cells were injected intravenously into C57BL/6 mice at day 10 post first CII immunization, followed by induction of CIA using the method described for **A**. Data are representative of two independent experiments with similar results, and are mean ± standard error of the mean. *P < 0.05, Student’s *t*-test.

To determine whether the suppressor cells regulate the development of arthritis in RORγt Tg mice, cells from draining LN from immunized C57BL/6 mice or RORγt Tg mice were adoptively transferred into immunized C57BL/6 mice, followed by induction of CIA. Interestingly, arthritis was significantly suppressed in mice recipient of cells from RORγt Tg mice but not those from C57BL/6 mice (Figure 4A). To identify the effectual cells, only CD4^+^ cells isolated from draining LN of C57BL/6 mice or RORγt Tg mice were adoptively transferred into immunized C57BL/6 mice. Arthritis was also suppressed in mice recipient of CD4^+^ cells isolated from RORγt Tg mice only, but not from C57BL/6 mice (Figure 4B). These results suggest that CD4^+^ T cells inhibit the development of autoimmune arthritis in RORγt Tg mice.

### RORγt overexpression augments the suppressive function of Foxp3^+^ Treg cells

**Figure 5:**
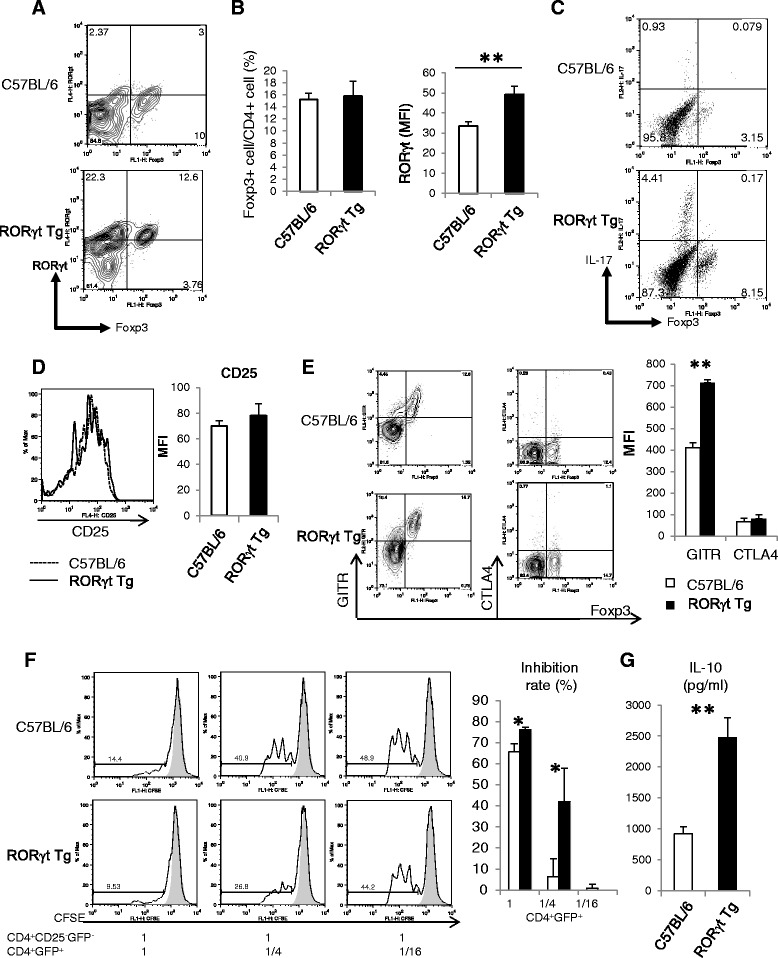
Overexpression of RORγt augments the suppressive capacity of Foxp3^+^ CD4^+^ Treg cells. **(A)** At day 10 post first CII immunization, the draining lymph-node (LN) cells were harvested from C57BL/6 mice (n = 3 or 4) and RORγt Tg mice (n = 3 or 4) and subjected to flow cytometry. **(B)** Percentage of Foxp3^+^ cells among CD4^+^ T cells and mean fluorescence intensity (MFI) of RORγt in Foxp3^+^ CD4^+^ cells. **(C)** Cells were analyzed by staining for intracellular IL-17 and Foxp3. Data represent gated CD4^+^. **(D)** Expression of CD25 on gated CD4^+^Foxp3^+^ cells. **(E)** Expression of surface markers glucocorticoid-induced tumor necrosis factor receptor (GITR) and cytotoxic T-lymphocyte-associated protein 4 (CTLA4) on Foxp3^+^CD4^+^ cells. Flow cytometric data (left) and MFI data of C57BL/6 mice and RORγt Tg mice. **(F)** Carboxyfluorescein diacetate succinimidyl ester (CFSE)-labeled CD4^+^CD25^−^GFP^−^ cells from C57BL/6-Foxp3^GFP^ mice cultured with or without CD4^+^GFP^+^ cells from C57BL/6-Foxp3^GFP^ mice or RORγt Tg-Foxp3^GFP^ mice and stimulated with anti-CD3/28 beads for 72 h. Left, representative data of two independent experiments with three mice per group; right, percentage of inhibition rate. **(G)** IL-10 production by CD4^+^ GFP^+^ cells of splenocytes stimulated for 72 h with anti-CD3/28 beads from C57BL/6-Foxp3^GFP^ and RORγt Tg-Foxp3^GFP^ mice. Representative data of two independent experiments with similar results, and are mean ± standard error of the mean values. *P <0.05, **P <0.01 versus C57BL/6 mice, Student’s *t*-test.

Foxp3-expressing CD4^+^ T cells are known to suppress T cell-mediated immune reaction [21]. In the next series of experiments, the expression of Foxp3 in CD4^+^ T cells was investigated by intracellular staining using LN cells harvested at day 10 post first CII immunization. Although there was no significant difference in Foxp3 expression in CD4^+^ T cells between RORγt Tg mice and C57BL/6 mice, higher expression of RORγt in Foxp3^+^ Treg cells was noted in RORγt Tg mice compared with C57BL/6 mice (Figure 5A, B).

To evaluate whether RORγt^+^ Foxp3^+^ Treg cells of RORγt Tg mice retain their stability and suppressive capacity, we analyzed the production of IL-17 and the expression of molecules known to be related to Foxp3^+^ Treg cell function. The FACS analysis of CD4^+^ T cells stimulated with CII *in vitro* showed IL-17 was produced only by Foxp3^−^ non-Treg cells in RORγt Tg mice (Figure 5C). The expression of CD25 (Figure 5D), which is associated with the stability of Foxp3 expression [22], was not significantly different between C57BL/6 and RORγt Tg mice. Although there was no difference in the expression of CTLA4, that of the co-inhibitory molecule, glucocorticoid-induced tumor necrosis factor receptor (GITR) was significantly higher in RORγt Tg than C57BL/6 mice (Figure 5E), suggesting that stability and suppressive capacity was not affected with overexpression of RORγt in Foxp3^+^ Treg cells.

To provide an answer to the question of whether Foxp3^+^ cells can suppress effector cell proliferation *in vitro*, we compared the ability of isolated CD4^+^GFP^+^ cells from C57BL/6-Foxp3^GFP^ and RORγt-Foxp3^GFP^ reporter mice to inhibit the proliferation of CD4^+^CD25^−^GFP^−^ cells from C57BL/6-Foxp3^GFP^ mice *in vitro*. Interestingly, the suppressive capacity of CD4^+^ GFP^+^ Treg cells was significantly enhanced in RORγt Tg Foxp3^GFP^ mice compared with C57BL/6-Foxp3^GFP^ reporter mice (Figure 5F), and CD4^+^GFP^+^ cells isolated from RORγt Tg Foxp3^GFP^ mice produced a larger amount of IL-10 than C57BL/6-Foxp3^GFP^ mice (Figure 5G). These results indicate that overexpression of RORγt seems to enhance the suppressive function of Foxp3^+^ Treg cells in RORγt Tg mice.

### RORγt overexpression enhances the chemotactic activity of Foxp3^+^ Treg cells

**Figure 6:**
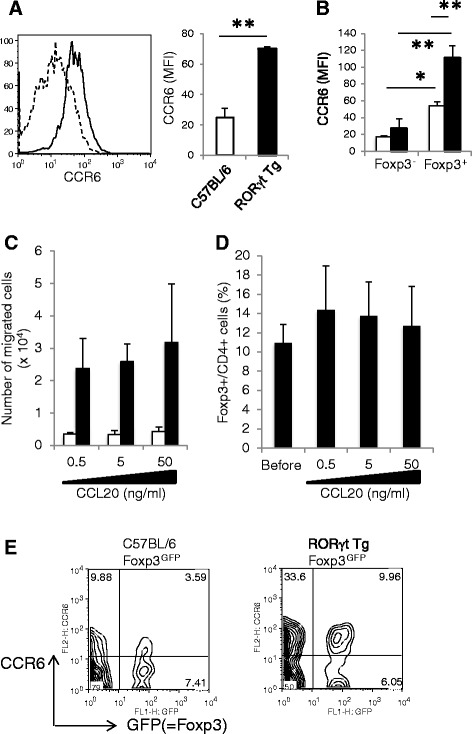
RORγt enhances chemotaxis of Foxp3^+^ Treg cells into inflamed tissues. **(A)** CCR6 expression in CD4^+^ T cells of C57BL/6 mice and RORγt Tg mice. **(B)** CCR6 expression in Foxp3^+^ CD4^+^ Treg cells and Foxp3^−^ CD4^+^ non-Treg cells from of C57BL/6 mice (open bars) and RORγt Tg mice (closed bars). **(C)** CD4^+^ cells were isolated by magnetic-activated cell sorting (MACS) from draining lymph-node (LN) cells of C57BL/6 mice (open bars) and RORγt Tg mice (closed bars), and added to the upper well of a Transwell system. The migration assay was performed in the presence of the indicated concentrations of CCL20 added to the bottom well. Data are numbers of respective cells that migrated to the bottom well counted by FACS. **(D) **CD4^+^ cells of RORγt Tg mice were subjected to intracellular Foxp3 staining, and then subjected to the migration analysis as in **C**. **(C** **,** **D)** Data are mean ± standard error of the mean. **P < 0.01, versus C57BL/6 mice, Student’s *t*-test. **(E)** Collagen-induced arthritis was induced in C57BL/6-Foxp3^GFP^ and RORγt Tg-Foxp3^GFP^ mice, and CD3^+^CD4^+^ T cells that infiltrated the ankle joints were harvested at day 30 post first type II collagen (CII) immunization and analyzed for green fluorescent protein (GFP) and CCR6 expression. Representative data of two independent experiments with similar results. MFI, mean fluorescence intensity.

Because CCR6 expression is positively regulated by RORγt and contributes to the recruitment of cells to the inflamed joints [14], we analyzed CCR6 expression on CD4^+^ T cells after CII immunization. CCR6 expression on CD4^+^ T cells was significantly higher in RORγt Tg than C57BL/6 mice (Figure 6A, *P* <0.01). Interestingly, the highest expression of CCR6 was observed in Foxp3^+^CD4^+^ Treg cells in RORγt Tg mice compared with Foxp3^+^CD4^+^ Treg cells in C57BL/6 mice and Foxp3^−^ non-Treg cells in RORγt Tg mice (Figure 6B).

To determine the chemotactic activity of CD4^+^ T cells to CCL20, the *in vitro* migration assay was performed using CCR6^+^ cells. More CD4^+^ cells migrated in response to CCL20 in RORγt Tg than C57BL/6 mice (Figure 6C). The migrating CD4^+^ T cells of RORγt Tg mice were enriched for Foxp3^+^ T cells (Figure 6D). In another *in vivo* experiment on mice, CIA was induced in both C57BL/6-Foxp3^GFP^ and RORγt Tg-Foxp3^GFP^ reporter mice, and the expression levels of CCR6 and GFP in CD4^+^ T cells infiltrating the joints were analyzed by flow cytometry. Similar to the results of *in vitro* migration assay, more CCR6^+^GFP^+^ Treg cells migrated into the joints of RORγt Tg-Foxp3^GFP^ reporter mice than C57BL/6-Foxp3^GFP^ reporter mice (Figure 6E). Considered together, these findings suggest that overexpression of RORγt induced CCR6 expression in Foxp3^+^ Treg cells and that the latter cells are enriched in the inflamed joints of RORγt Tg mice.

### Neutralization of IL-10 exacerbates CIA in RORγt Tg mice

**Figure 7:**
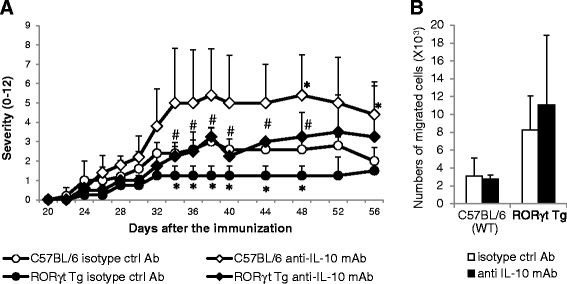
Neutralization of IL-10 exacerbates collagen-induced arthritis in RORγt Tg mice. **(A)** C57BL/6 mice (n = 5) and RORγt Tg mice (n =4) were immunized intradermally with chicken type II collagen (CII) emulsified with complete Freund’s adjuvant (CFA) on days 0 and 21. Anti-IL-10 monoclonal antibody (mAb) or isotype control antibody (ctrl Ab) were administrated intraperitoneally every 2 days from day 22 to 30 post first CII immunization. Severity of arthritis was shown. Representative data are mean ± standard error of the mean (SEM) values. *P <0.05 versus C57BL/6 mice with the administration of isotype ctrl Ab, ^#^ *P <*0.05 versus RORγt Tg mice with the administration of isotype ctrl Ab, Student’s *t*-test. **(B)** At day 10 post first CII immunization, CD4^+^ cells were isolated by magnetic-activated cell sorting (MACS) from draining lymph-node cells of C57BL/6 mice (open bars) and RORγt Tg mice (closed bars), and added to the upper well of a Transwell system. The migration assay was performed in the presence of CCL20 and anti-IL-10 mAb or isotype ctrl Ab added to the bottom well. Data are number of respective cells that migrated to the bottom well counted by FACS. Representative data of two independent experiments with similar results and are mean ± SEM value.

To clarify whether the suppression of CIA is related to IL-10, we administrated anti-IL-10 monoclonal antibody (mAb) to neutralize IL-10 in the course of CIA. The severity of arthritis was significantly exacerbated in both C57BL/6 mice and RORγt Tg mice administrated with anti-IL-10 mAb compared with the mice with isotype control antibody, respectively (Figure 7A). However, suppression of CIA was not completely cancelled in RORγt Tg mice with the administration of anti-IL-10 mAb, although there was no statistically significant difference between C57BL/6 mice and RORγt Tg mice treated by anti-IL-10 mAb (Figure 7A). We also examined the effect of IL-10 neutralization on the chemotactic activity of CD4^+^ T cells isolated from draining LNs at day 10 post first CII immunization *in vitro*. There was no significant difference in the migration of CD4^+^ T cells in response to CCL20 by the addition of anti-IL-10 mAb in both two groups of mice (Figure 7B). These data indicated that IL-10 is related to the inhibition of the development of CIA in RORγt Tg mice.

## Discussion

The transcription factor RORγt is a master regulator of the differentiation of Th17 cells and expression of Th17 cytokines; IL-17A, IL-17 F, IL-22 and IL-21 [13]. Previous studies indicate that both IL-17 and Th17 cells seem to be involved in the pathogenesis of rheumatoid arthritis and animal model of autoimmune arthritis [3-7,17]. We have previously reported that the development of murine autoimmune arthritis is suppressed by the overexpression of T-bet, a master regulator of differentiation of Th1 cells, and suggested that Th17 cells differentiation is inhibited by T-bet through downregulation of RORγt [16]. To elucidate the effects of RORγt on T cell function in autoimmune arthritis, we used RORγt Tg mice under the control of CD2 promoter. In RORγt Tg mice, most T cells were considered Th17 cells based on the high expression level of RORγt and production of high amounts of IL-17, compared to comparable levels of IFNγ and IL-4 found in WT mice [16]. Contrary to our expectation, overexpression of RORγt provided protection against the development of autoimmune arthritis in mice. While the exact mechanism of this protection is unknown, we propose three scenarios: imbalance in Th1/Th17 cell ratio, low anti-CII antibody formation, and CD4^+^ Treg cell hyperfunction.

Does imbalance in the Th17/Th1 cell ratio play a pathogenic role in experimentally induced CIA in RORγt Tg mice? Previous studies reported that Th1 cells have anti-inflammatory properties in experimental arthritis [16,23,24]. Accordingly, we focused in this study on cytokine production and transcription factor expression in CII reactive CD4^+^ T cells. The results showed significantly high IL-17 production and RORγt expression in CII-reactive T cells of RORγt Tg mice, but no differences in IFNγ production and T-bet expression, compared with C57BL/6 mice. These findings argue against the Th17/Th1 cell imbalance theory as an explanation for the suppression of CIA in RORγt Tg mice.

Is anti-CII antibody titer important in the development of CIA in RORγt Tg mice? A previous study showed that the development of CIA correlates well with the level of serum anti-CII antibody, particularly the IgG2 subclass [20]. The significantly low level of serum anti-CII total IgG could be one of the causes for the suppression of CIA in RORγt Tg mice, although there was no significant difference in CII-specific IgG2 subclass between the two types of mice. Leavenworth *et al*. [25] reported recently that Tfh-dependent autoantibody production results in immune complex formation in joint tissues, and complement activation, and that enhanced intra-articular inflammatory responses induced by Th17 cells play a role in the development of autoimmune arthritis. Tfh cells are known to express CXCR5, ICOS, and PD-1 as superficial markers, and to produce IL-21 for help for B cells [26-28]. In addition, lineage commitment of Tfh cells is directly regulated by transcription factor Bcl-6 [28]. Our results showed lower expression levels of Bcl-6 and IL-21 in CXCR5^+^CD4^+^ Tfh cells in RORγt Tg mice than C57BL/6 mice, although the ICOS^+^PD-1^+^ Tfh cells increased in draining LNs of RORγt Tg mice, suggesting that the differentiation and the function of Tfh cells might be suppressed in RORγt Tg mice, and which is associated with the reduced anti-CII antibody formation and the suppression of the development of CIA. Although the precise mechanism was not elucidated, the low level of anti-CII antibody might be also related to the diminished ectopic lymphoid follicle formation induced by local synovial inflammation, which is distinct feature of autoimmune arthritis [29,30].

The third possible etiology of arthritis in RORγt Tg mice involves overexpression of RORγt. This scenario is based on the attenuation of CIA in not only RORγt Tg mice but also in C57BL/6 mice with adoptive transfer of CD4^+^ T cells from RORγt Tg mice. Forkhead family transcription factor Foxp3 is characteristically expressed in a major subset of regulatory T cells [31]. Foxp3^+^ Treg cells can suppress the activation of Th17 cells and other effector T cell subsets as well as the development of certain autoimmune diseases, including CIA [32]. Interestingly, RORγt expression on Foxp3^+^ Tregs was significantly upregulated in RORγt Tg mice compared with C57BL/6 mice, although there were no difference between the two groups of mice in Foxp3 expression on CD4^+^ T cells. Foxp3^+^ Treg cells also expressed high levels of the co-inhibitory molecule, GITR [33], produced high amounts of IL-10 but not IL-17, and suppressed the proliferation of effector T cells in RORγt Tg mice. Moreover, the suppression of CIA was partially cancelled by the neutralization of IL-10 in RORγt Tg mice, suggesting that IL-10 produced from Foxp3^+^ Treg cells might be related to attenuation of CIA in RORγt Tg mice. In addition to our observations, Lochner *et al*. reported that RORγt^+^ T cells include pro-inflammatory IL-17-producing Th17 cells and IL-10-producing Foxp3^+^ Treg cells, and that equilibrium of two types of RORγt^+^ cells are tightly controlled *in vivo* [34]. Although the precise mechanism of enhanced suppressive capacity of Foxp3^+^ Treg cells in RORγt Tg mice was not elucidated, these data support our hypotheses that IL-10-producing RORγt^+^Foxp3^+^ Treg cells suppress the development of CIA.

Previous studies report that RORγt can induce the expression of CCR6, which is also known to play a role in arthritogenic Th17 cell recruitment to inflamed joints [14]. Furthermore, CCR6-expressing Treg cells can reduce the Th17-mediated inflammatory response [35,36]. In the present study, RORγt upregulation induced overexpression of CCR6 in Foxp3^+^ Treg cells, which in turn resulted in preferential migration of Foxp3^+^ Treg cells in response to CCL20, a ligand of CCR6. Although we have no direct evidence for the involvement of Foxp3^+^ Treg in the pathogenesis of CIA, the results of previous studies and our findings suggest upregulation of RORγt enhances the expression of CCR6 on Foxp3^+^ Treg cells, resulting in preferential infiltration of Foxp3^+^ Treg cells into inflamed joints and suppression of autoimmune synovitis.

## Conclusion

Our results suggest that the protective effects of RORγt overexpression against the development of CIA in mice were mediated through the anti-inflammatory effects of intra-articular IL-10-producing CCR6^+^RORγt^+^Foxp3^+^ Treg cells. The results also suggest that modulation of transcription factor expression on CD4^+^ T cells is a potentially useful therapeutic approach in RA.

## Abbreviations

Bcl-6: B cell lymphoma 6
CCL20: CC chemokine ligand 20
CCR6: CC chemokine receptor 6
cDNA: complementary deoxyribonucleic acid
CFA: complete Freund’s adjuvant
CFSE: carboxyfluorescein diacetate succinimidyl ester
CIA: collagen induced arthritis
CII: type II collagen
CTLA-4: cytotoxic T-lymphocyte-associated protein 4
CXCR5: CXC chemokine receptor 5
ELISA: enzyme-linked immunosorbent assay
FACS: fluorescence-activated cell sorting
FBS: fetal bovine serum
Foxp3: forkhead box P3
GATA-3: GATA binding protein-3
GFP: green fluorescent protein
GITR: glucocorticoid-induced tumor necrosis factor receptor
GPI: glucose-6-phosphate isomerase
ICOS: inducible T-cell do-stimulator
IFNγ: interferon γ
IL: interleukin
LN: lymph node
mAb: monoclonal antibody
MACS: magnetic-activated cell sorting
MFI: mean fluorescence intensity
PBS: phosphate-buffered saline
PCR: polymerase chain reaction
PD-1: programmed cell death-1
PI: propidium iodide
RA: rheumatoid arthritis
RNA: ribonucleic acid
RORγt Tg: RORγt transgenic
RORγt: retinoid-related orphan receptor gamma t
RT: reverse transcriptase
SEM: standard error of the mean
T-bet: T-box transcription factor
Tfh: T follicular helper
TGF-β: transforming growth factor-β
Th: T helper
Treg: regulatory T
WT: wild-type
